# The association of the age, period, and birth cohort with 15-year changes in body mass index and waist circumference in adults: Tehran lipid and glucose study (TLGS)

**DOI:** 10.1186/s12889-022-12810-z

**Published:** 2022-03-02

**Authors:** Maryam Barzin, Shayan Aryannezhad, Mohammad Bagheri, Maryam Mahdavi, Majid Valizadeh, Fereidoun Azizi, Farhad Hosseinpanah

**Affiliations:** 1 Obesity Research Center, Research Institute for Endocrine Sciences, Shahid Beheshti University of Medical Sciences, Tehran, Iran; 2 Endocrine Research Center, Research Institute for Endocrine Sciences, Shahid Beheshti University of Medical Sciences, Tehran, Iran

## Abstract

**Objective:**

To examine the association of age, period, and birth cohort with body mass index (BMI) and waist circumference (WC) changes among the participants of the Tehran Lipid and Glucose Study from 1999 to 2015.

**Methods:**

This prospective cohort study included 4895 participants aged ≥20 years (41.3% men), who were divided into twelve gender stratified groups, having a ten-year age difference between them. Analyses were conducted to explicitly assess the association of age vs. period on BMI and WC changes. In addition, we evaluated BMI and WC changes among different birth cohorts.

**Results:**

Upon 15 years of follow-up, the mean BMI of men and women increased from 26.0 ± 3.9 to 27.5 ± 4.3 kg/m^2^ and from 27.5 ± 4.8 to 29.9 ± 5.4 kg/m^2^ (P trend < 0.001), and this trend was accompanied by an increase in WC from 88.8 ± 10.9 to 97.8 ± 10.4 cm and from 87.3 ± 12.4 to 95.8 ± 12.1 cm, respectively (P trend < 0.001). Men and women in all age cohorts tended to have a rise in their BMI and WC with aging throughout the follow-up period. For men, this trend was more prominent in younger birth cohorts at phase III for BMI and at phases III and V for WC (indicating a significant negative association with birth cohort). For women, this trend was more prominent in older birth cohorts at both phases III and V for BMI and WC (indicating a significant positive association with the birth cohort).

**Conclusion:**

The rise in BMI and WC was strongly associated with age in both sexes. The men born in the recent birth cohorts and the women born in earlier birth cohorts had the most alarming BMI and WC trends. More efforts must be spent on obesity prevention policies, especially for younger men.

**Supplementary Information:**

The online version contains supplementary material available at 10.1186/s12889-022-12810-z.

## Background

Obesity is highly prevalent in developed and developing countries [1–3]. In the recent decades, Iran, as a developing country, has been experiencing an alarming rise in the prevalence of obesity; over an 8-year period, the overall prevalence of obesity in the country increased from 13.6% in 1999 to 22.3% in 2007 (OR = 1.08 per year) [4]. Increasing trends in general and abdominal obesity have been reported in both genders in Iran’s capital, Tehran. This trend has contributed to an overall 36 and 34% increased risk of general and abdominal obesity from 1999 to 2011, respectively [5].

Various patterns in trends of obesity can be explainable by many sociodemographic, socioeconomic, and lifestyle factors. To explore these patterns, it is necessary to assess overtime changes in measures of general obesity (e.g., body mass index [BMI]) and abdominal obesity (e.g., waist circumference [WC]) in individuals (i.e., the age effect); and based on population-wide behavioral changes and other age-independent exposures (i.e., the period effect); as well as based on intra-individual differences in the people born in different times (i.e., the birth-cohort effect) [6]. Rising trends in BMI and WC with aging, as observed in cross-sectional studies, could be the result of the age and birth-cohort effects. On the other hand, increases in BMI and WC with aging, as reported in longitudinal studies, might be due to period effects. The simultaneous assessment of the age, period, and birth-cohort effects on BMI and WC require longitudinal analyses with repeated measures in the same individuals [7–9].

Over the last few decades, Iran has experienced an epidemiological transition from a traditional to a western lifestyle [10]. There are no recent studies evaluating age and/or cohort effects on BMI and WC based on repeated measurements in Iran. Here, we investigated the association of age, period, and birth-cohort with BMI and WC changes in the framework of a prospective cohort study within a 15-year follow up period.

## Material and methods

### Study population

The Tehran Lipid and Glucose Study (TLGS) is a longitudinal cohort, which commenced in 1999 with the objective of determining the prevalence of non-communicable diseases among urban residents of Tehran, as a representative sample of the total population of Iran’s capital. A multistage stratified cluster random sampling technique was used to select 15,010 people aged ≥3 years old. From this population, only individuals above 20 years of age who participated in phase I (1999-2001), phase III (2006-2008), and phase V (2012-2015) of the study were enrolled. The intervals between each two assessments (from phase III to phase I and from phase V to phase III) were approximately six years. The details of the study have been published elsewhere [11]. After excluding the subjects aged <20 years (*n*=4647), those with cancer (*n*=44), pregnant participants (*n*=80), the patients using glucocorticoids (*n*=211), and the participants who lost the follow-up or had not participated in all the three phases (*n*=5133), the data of 4895 subjects were used in this longitudinal study. These individuals included 2024 men (41.3%) and 2871 women (58.7%) who attended phases I, III, and V of TLGS with a median follow-up of 13.0 (IQ: 12.3-13.9) years. This study was approved by the Research Ethics Committee of the Research Institute for Endocrine Sciences, Shahid Beheshti University of Medical Sciences, and informed written consent was obtained from all subjects. All procedures were conducted in accordance with the principles of the Declaration of Helsinki.

### Measurements and definitions

Weight and height were determined using a digital electronic weighing scale (Seca 707; range: 0.1–150 kg, Hanover, MD, accuracy of up to 100 g) and a tape meter stadiometer, respectively. Waist circumference was measured at the level of the umbilicus by a trained individual, and BMI was calculated as weight (kg)/height (m^2^) [12]. Educational level was categorized into ≤12-year education (primary school, secondary school, and high-school diploma) and > 12 years of education (i.e., university level). Smoking status was categorized into yes/no, ‘Yes’ meaning tobacco smoking (cigarette, pipe, or water pipe) at the time of examination and ‘no’ as no smoking at the time (ex- or never-smokers). Information on leisure-time physical activity (LTPA) were collected using a Persian-translated form of the Modifiable Activity Questionnaire (MAQ) [11], which was categorized into the light or non-active (MET < 600 min/week) and moderate/vigorous or active (MET ≥600 min/week) groups [13]. Marital status was regarded as either married or unmarried (including never-married, widowed, and divorced).

### Statistical analysis

Continuous variables were expressed as mean ± SD and categorical variables as number (percent). These variables were compared between respondents and non-respondents using *t*-test for continuous variables and Chi square for categorical variables. A series of repeated measures models were used to assess differences in anthropometric factors between the three phases. Differences in categorical factors (i.e., smoking, physical activity, marital status, and education) along the follow-up period were analyzed using the generalized estimated equation (GEE) method with an autoregressive working correlation structure and a logistic model. Post-hoc Bonferroni correction was used for pairwise comparisons. Analyses were stratified by sex and age group. Mixed effects models with fixed and random individual-level effects and random slopes were used to assess differences in anthropometric factors among individuals over time. The stratified models included age and age-squared, as well as age interaction with year to determine how the survey year may vary by age. Figures were stratified by age groups to provide insights into the period-specific association of covariates. All analyses were performed in the SPSS statistical software package (SPSS for Windows; SPSS Inc., Chicago, IL, USA; Version 20.00), and mixed effects models were conducted using Stata’s XTMIXED program. Statistical significance was considered two-tailed.

## Results

The mean age of study participants at baseline was 41.4 ± 13.0 years, and 58.7% of the participants were women. After 15 years of follow-up, mean BMI in men increased from 26.0 ± 3.9 to 27.5 ± 4.3 kg/m^2^ (P _trend_ < 0.001), and this trend was accompanied by an increase in mean WC from 88.8 ± 10.9 to 97.8 ± 10.4 cm (P _trend_ < 0.001). Women also showed elevations in mean BMI (from 27.5 ± 4.8 to 29.9 ± 5.4 kg/m^2^) and mean WC (from 87.3 ± 12.4 to 95.8 ± 12.1 cm) in this period (P _trend_ < 0.001). More detailed characteristics of the study population across different phases of the study are available in Table 1. In comparison with those who completed the follow-up and entered the study (the respondent group, *n* = 4895), non-respondents (*n* = 5133) were older and had lower BMIs (supplementary Table 1).

**Table 1:** Sex-specific characteristics of study population across phases I, III, and V

	Phase I	Phase III	Phase V	***P***_**I-III**_^a^	***P***_**III-V**_	***P***_**I-V**_
**Men** (*N* = 2024)						
** Weight** (kg)	75.2 ± 12.3	78.9 ± 13.2	80.5 ± 14.3	< 0.001	< 0.001	< 0.001
** WC** (cm)	88.8 ± 10.9	96.4 ± 10.1	97.8 ± 10.4	< 0.001	< 0.001	< 0.001
** BMI** (kg/m^2^)	26.0 ± 3.9	27.2 ± 4.0	27.5 ± 4.3	< 0.001	< 0.001	< 0.001
** Smoking** (non-smokers)	1516 (75.4)	1549 (77.8)	1609 (79.7)	0.006	0.011	< 0.001
** Physical activity** (active) ^b^	408 (20.3)	228 (19.6)	208 (19.4)	0.431	> 0.999	> 0.999
** Marital status** (married)	1698 (83.9)	1903 (94.2)	1966 (97.2)	< 0.001	< 0.001	< 0.001
** Education** (> 12 years)	374 (18.5)	488 (24.5)	545 (26.9)	< 0.001	< 0.001	< 0.001
**Women** (*N* = 2871)						
** Weight** (kg)	67.6 ± 12.0	70.2 ± 12.1	71.7 ± 12.6	< 0.001	< 0.001	< 0.001
** WC** (cm)	87.3 ± 12.4	90.8 ± 12.6	95.8 ± 12.1	< 0.001	< 0.001	< 0.001
** BMI** (kg/m^2^)	27.5 ± 4.8	28.9 ± 4.9	29.9 ± 5.4	< 0.001	< 0.001	< 0.001
** Smoking** (non-smokers)	2783 (97.6)	2731 (97.3)	2771 (96.9)	0.978	0.348	0.058
** Physical activity** (active) ^b^	760 (26.7)	240 (13.8)	166 (12.9)	< 0.001	> 0.999	< 0.001
** Marital status** (married)	2597 (90.5)	2732 (95.2)	2758 (96.2)	< 0.001	< 0.001	< 0.001
** Education** (> 12 years)	251 (8.8)	377 (13.4)	449 (15.7)	< 0.001	< 0.001	< 0.001

Data are presented as mean ± SD or n (%)

*WC* waist circumference; *BMI* body mass index

^a^Adjustment for multiple comparisons with Bonferroni correction (comparisons with phase1 and phase3)

^b^Active Physical activity was defined as MET ≥600 min/week by using the Modifiable Activity Questionnaire

**Fig. 1:**
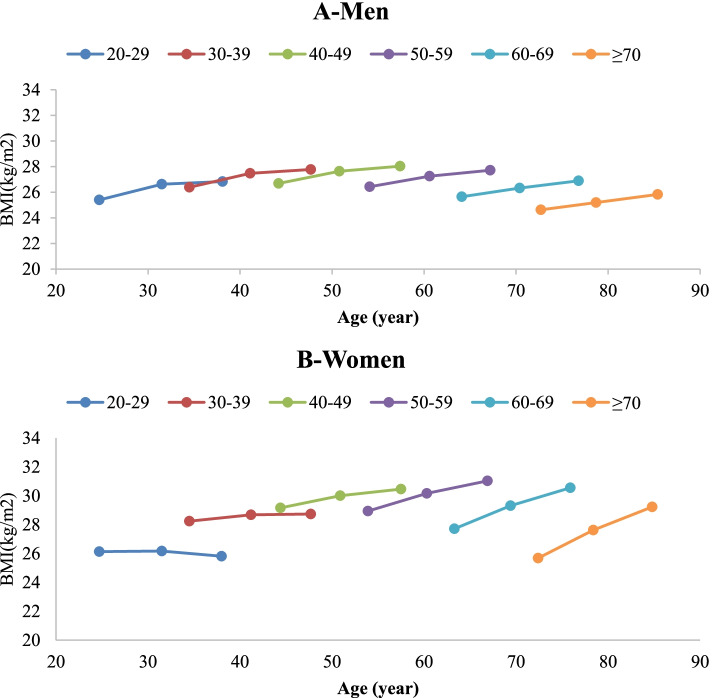
Trends of body mass index (BMI) in different age cohorts of study population across phases I, III, and V; in men (**A**) and women (**B**)

**Fig. 2:**
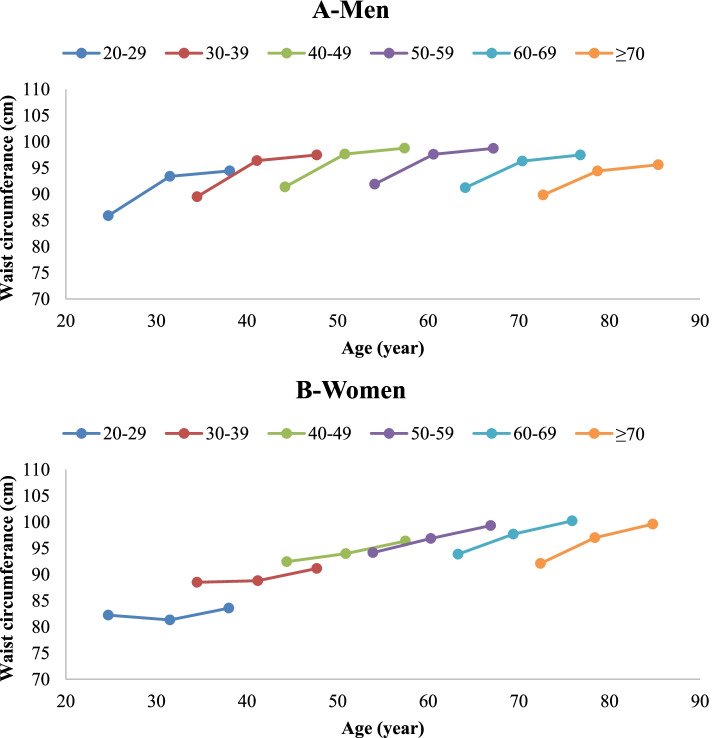
Trends of waist circumference in different age cohorts of study population across phases I, III, and V; in men (**A**) and women (**B**)

Cohort-specific mean BMI and WC values in the study population are presented in Table 2. Rising trends in mean BMI and WC in each age cohort were observed for both genders (except for the BMI of ≥70 years old in both genders and WC of ≥70 years old women). Additionally, sex-specific mixed effects were estimated on BMI and WC changes in the studied population, adjusted for age, age squared, time, time*age, smoking, physical activity, education, and marital status (Tables 3 and 4). In Figs. 1 and 2, adjusted estimated means of BMI and WC in six age cohorts are shown according to the mean age of the group in each phase of the study, in men and women.

**Table 2:** Sex-specific body mass index (BMI) and waist circumference of different age cohorts of study population across phases I, III, and V

			BMI (kg/m^**2**^)				Waist Circumference (cm)			
	Age cohort (years)	N	Phase I	Phase III	Phase V	***P***_trend_	Phase I	Phase III	Phase V	***P***_trend_
**Men**	**20–29**	371	24.5 ± 4.3	27.2 ± 4.3	28.1 ± 4.3	< 0.001	82.5 ± 11.4	95.3 ± 10.9	98.4 ± 10.7	< 0.001
	**30–39**	576	26.2 ± 3.9	27.6 ± 4.3	28.1 ± 4.4	< 0.001	88.1 ± 10.5	96.3 ± 10.5	98.4 ± 10.8	< 0.001
	**40–49**	442	26.4 ± 3.5	27.4 ± 3.7	27.5 ± 4.2	< 0.001	90.3 ± 9.7	96.7 ± 9.3	97.7 ± 9.5	< 0.001
	**50–59**	336	26.7 ± 3.6	27.1 ± 3.6	27.1 ± 3.8	0.001	92.2 ± 9.9	97.4 ± 9.3	98.1 ± 9.9	< 0.001
	**60–69**	250	26.2 ± 3.8	26.4 ± 3.7	26.0 ± 3.9	0.005	92.0 ± 10.7	96.9 ± 10.3	96.1 ± 10.9	< 0.001
	**≥70**	49	25.6 ± 3.6	25.8 ± 3.9	25.5 ± 4.5	0.395	90.4 ± 8.8	95.8 ± 9.6	95.5 ± 11.6	< 0.001
	**Total**	2024	23.4 ± 5.4	25.9 ± 4.7	27.0 ± 4.5	< 0.001	88.8 ± 10.9	96.4 ± 10.10	97.8 ± 10.1	< 0.001
**Women**	**20–29**	618	24.4 ± 4.6	26.5 ± 4.7	28.0 ± 4.9	< 0.001	78.3 ± 11.3	82.1 ± 11.5	89.0 ± 10.9	< 0.001
	**30–39**	816	27.1 ± 4.5	28.7 ± 4.8	29.9 ± 5.0	< 0.001	84.7 ± 11.0	88.5 ± 11.5	94.2 ± 11.4	< 0.001
	**40–49**	706	29.2 ± 4.4	30.4 ± 4.6	31.3 ± 6.0	< 0.001	91.3 ± 11.1	94.8 ± 11.3	99.1 ± 11.5	< 0.001
	**50–59**	496	29.4 ± 4.3	30.2 ± 4.6	30.9 ± 4.9	< 0.001	93.9 ± 10.5	97.3 ± 11.0	100.7 ± 11.2	< 0.001
	**60–69**	215	28.4 ± 4.1	29.1 ± 4.3	29.3 ± 4.7	< 0.001	94.5 ± 10.2	96.7 ± 10.9	98.8 ± 11.5	< 0.001
	**≥70**	20	27.3 ± 3.4	27.8 ± 3.1	27.2 ± 3.7	0.418	92.1 ± 9.7	93.6 ± 7.6	94.3 ± 9.7	0.545
	**Total**	2871	25.0 ± 6.2	27.2 ± 5.6	28.6 ± 5.6	< 0.001	87.3 ± 12.4	90.8 ± 12.6	95.8 ± 12.1	< 0.001

Data are presented as mean ± SD

**Table 3:** Sex-specific mixed effects estimates on body mass index (BMI) of study population, coefficient (95% CI)

	Coefficient (95% CI)	***P***-value
**Men**		
Age	0.28(0.20, 0.35)	< 0.001
Age^2^	−0.003(− 0.004, − 0.002)	< 0.001
Time (Ref: Phase I)		
Phase III	1.68(1.06, 2.30)	< 0.001
Phase V	1.60(0.41, 2.78)	0.008
Time*age (Ref: Phase I)		
Phase III *age	−0.014(− 0.027, − 0.002)	0.028
Phase V *age	− 0.005(− 0.028, 0.018)	0.662
Smoking (Ref: non-smoker)	− 0.38(− 0.57, − 0.19)	< 0.001
Physical activity (Ref: high)	0.16(0.05, 0.27)	0.005
Education (Ref: > 12 year)	−0.07(− 0.31, 0.16)	0.534
Spouse: (Ref: married)	−1.03(−1.30, − 0.77)	< 0.001
**Women**		
Age	0.61(0.53, 0.69)	< 0.001
Age^2^	−0.006(− 0.007, − 0.005)	< 0.001
Time (Ref: Phase I)		
Phase III	−1.24(− 1.89, − 0.59)	< 0.001
Phase V	−2.92(−4.20, − 1.65)	< 0.001
Time*age (Ref: Phase I)		
Phase III *age	0.04(0.03, 0.05)	< 0.001
Phase V *age	0.08(0.06, 0.11)	< 0.001
Smoking (Ref: non-smoker)	−0.69(−1.14, − 0.23)	0.003
Physical activity (Ref: high)	0.12(0.02, 0.23)	0.024
Education (Ref: > 12 year)	0.74(0.46, 1.03)	< 0.001
Marital status: (Ref: married)	−1.69(−2.03, − 1.35)	< 0.001

Model includes age, age-squared, study phase year, age interacted with study phase year, smoking, education, and marital status

**Table 4:** Sex-specific mixed effects estimates on waist circumference of study population, coefficient (95% CI)

	Coefficient (95% CI)	***P***-value
**Men**		
Age	0.77(0.57, 0.96)	< 0.001
Age^2^	−0.006(− 0.008, − 0.004)	< 0.001
Time (Ref: Phase I)		
Phase III	9.49 (7.88, 11.10)	< 0.001
Phase V	10.44(7.39, 13.49)	< 0.001
Time*age (Ref: Phase I)		
Phase III*age	−0.06(−0.10, − 0.03)	< 0.001
Phase V*age	−0.06(− 0.12, − 0.001)	0.045
Smoking (Ref: non-smoker)	−1.12(−1.63, − 0.61)	< 0.001
Physical activity (Ref: high)	0.77(0.48,1.07)	< 0.001
Education (Ref: > 12 year)	−0.33(− 0.95, 0.29)	0.295
Spouse: (Ref: married)	−3.11(−3.83,-2.40)	< 0.001
**Women**		
Age	1.32(1.14, 1.50)	< 0.001
Age^2^	−0.010(− 0.013,-0.008)	< 0.001
Time (Ref: Phase I)		
Phase III	−4.87(−6.57, −3.17)	< 0.001
Phase V	−2.84(−5.90, 0.23)	0.070
Time*age (Ref: Phase I)		
Phase III*age	0.13(0.09, 0.16)	< 0.001
Phase V*age	0.13(0.07, 0.19)	< 0.001
Smoking (Ref: non-smoker)	−0.76(−2.12, 0.60)	0.274
Physical activity (Ref: high)	0.80(0.46, 1.15)	< 0.001
Education (Ref: > 12 year)	2.63(1.83, 3.42)	< 0.001
Spouse: (Ref: married)	−5.25(−6.26,-4.24)	< 0.001

Model includes age, age-squared, study phase year, age interacted with study phase year, smoking, education, and marital status

### Subgroup gender analysis

Table 3 and Table 4 show sex-specific mixed effects models on BMI and WC of study participants, adjusted for confounding variables. Un-adjusted models also revealed the same statistical results, though the data is not shown. Coefficient with a 95% confidence interval (95% CI) was calculated for various variables of the adjusted model. For the age variable, the coefficient (95% CI) represents the changes in the BMI or WC contributed to a 1-year increase in age of participants throughout the study. For the time variable, the coefficient (95% CI) represents the changes in the BMI or WC contributed to change in time (Phase V or III in reference to phase I). Time*age indicates the interaction of these two variables, representing the changes in the BMI or WC contributed to change in time, independent of changes in age. In brief, the adjusted models showed men in all age cohorts had a rise in BMI with aging throughout the follow-up period (coefficient for age: 0.28, 95% CI: 0.20, 0.35). However, as shown in Fig. 1-A, this rise was more prominent in younger cohorts at phase III (coefficient for time*age: − 0.014, 95% CI: − 0.027, − 0.002), indicating a significant negative association with birth cohort. Considering WC changes, all age cohorts had a rising trend with aging throughout the follow-up (coefficient for age: 0.77, 95% CI: 0.57, 0.96). Significant negative association with birth cohort were observed at phases III and V (coefficient for time*age: − 0.06, 95% CI: − 0.10, − 0.03 and − 0.06, 95% CI: − 0.12, − 0.001, respectively) (Fig. 2-A). Moreover, it was observed that higher physical activity, smoking, lower education, and not being married were associated with lower weight and WC.

Women in all age cohorts showed a rise in BMI throughout the follow-up with aging (coefficient for age: 0.61, 95% CI: 0.53,0.69). However, this trend was influenced by a positive association with birth cohort at both phases III and V (coefficient for time*age 0.04, 95% CI: 0.03, 0.05 and 0.8, 95% CI: 0.06, 0.11, respectively), meaning that older age cohorts were more likely to gain weight with time (Fig. 2-A). Regarding WC changes, all age cohorts displayed a rising trend throughout the follow-up period with aging (coefficient for age: 1.32, 95% CI: 1.14, 1.50). In line with BMI changes, this trend was influenced by a positive association with birth cohort at both phases III and V (coefficient for time*age 0.13, 95% CI: 0.09, 0.16 and 0.13, 95% CI: 0.07, 0.19, respectively) (Fig. 2-B). Regarding the association of other variables, higher physical activity, smoking, a higher education, and not being married were associated with lower weight and WC.

## Discussion

In this 15-year population-based longitudinal study, gender-specific changes of BMI and WC in the adult population of Tehran were evaluated. The rises in BMI and WC were strongly associated with aging throughout the follow up period. Increases in BMI and WC were more prominent in younger birth cohorts in men, and older birth cohorts in women.

A large body of evidence suggests that weight, BMI, and WC increase throughout most of adult life [14–17], which is in line with our findings regarding the positive association of aging with obesity measures, observed in both genders. A prospective longitudinal study in Norway recently showed that mean weight in middle-aged men and women (aged 26–54 years) increased significantly during a 10-year follow-up while it reduced in elderly men and women (aged 60–69 years); however, mean WC increased throughout the follow up in all age groups [18]. The recent findings were completely in accordance with those of the present study. We observed an overall positive association of aging with BMI in both genders which in combination with the observed inverse association of BMI with age squared suggested an inverted U-shaped relationship between age and weight gain. This implies that the rapid weight gain seen in young and middle-aged men and women will most likely be reversed in the elderly. The weight reduction with aging reported in the elderly could be a result of decreased appetite and calorie intake, as well as substantial loss of muscle mass due to the aging process [19]. Apart from age-related muscle loss, disease-related muscle loss could also explain this phenomenon, as many chronic diseases (with particularly higher prevalence in older individuals) could also accelerate decrease of muscle mass, such as diabetes, hypogonadism in men, growth hormone deficiency, hyperthyroidism, and hypercortisolism [20]. However, in older men and women, despite a drop in BMI, WC increased over years, which could be a result of body composition changes. Age has been shown to affect body composition with older people having higher proportions of the adipose tissue to the lean mass [21]. Furthermore, age affects the distribution of the adipose tissue, with more intra-abdominal and visceral adipose tissues vs. peripheral subcutaneous fat mass being observed in the elderly [22]. Besides, spinal degenerative changes and a consequent reduction in height during the aging process could explain the increase of WC despite decreased or sustained weight in this age group [23].

In this study, a significant negative association of birth cohort with BMI and WC was observed in adult men, indicating notable general and central weight gain in the youngest compared with older birth cohorts. This finding was consistent with the observations of previous studies [7, 9, 24–30]. Iran, as a country located in the Middle East and North Africa region, is subjected to a dramatic epidemiological transition from a traditional to modern nutrition and lifestyle [10]. This modernization and associated lifestyle changes by encouraging sedentary habits and excess calorie intake have increased the risk of obesity and overweight in the new generations of Iranians, and as a consequence, the prevalence of obesity among children and adolescents has increased [31–33]. Overweight or obese children and adolescents are likely to remain so in adulthood, and this may explain why people who were born in the most recent birth cohorts were more susceptible to BMI and WC elevations than older individuals born in the years of economic and social instability. Moreover, in older men, this negative association of birth cohort could be partially explained by reduced calorie intake and aging-associated loss of appetite [19]. In addition, exposure to stress might lead to unhealthy behaviors which could contribute to weight gain as well [34]. Another explanation for the observed association of weight gain with younger birth cohorts could be more job commitments of younger generations and their less time for physical exercises and self-care, delivering them more prone to obesity. In contrast, the individuals born in earlier birth cohorts spent their youth in more active lifestyles and were less exposed to cumulative obesogenic health behaviors [35, 36]. Another explanation for the negative impact of birth cohort on men could be related to the survival bias which might be a consequence of premature mortality among older obese individuals [37]. In contrast to our findings, a study on Chinese adults found that being overweight was more common among middle-aged men born between 1950 and 1975 and revealed that the prevalence of overweight among Chinese adults decreased rapidly in the most recent birth cohorts (i.e., after 1975) [38]. The observed difference between the two studies may be due to economic and social differences between the two populations. China has become one of the world’s fastest growing economies since the 1980s and with increased access to health information and the implantation of new health policies, has somehow managed to overcome the epidemiological transition [39].

In women, however, we observed a positive association of birth cohort with BMI. Regarding the difference between men and women in this area, both biological and social factors may be accountable for justifying the reported disparity between the two genders. The younger women born in recent birth cohorts are more likely to have higher education, have fewer pregnancies, and adhere to feminine beauty standards paying a premium for slimness as a desirable body form [40]. Moreover, menopausal changes affect women in their later years, making elderly women more prone to obesity [41, 42]. Another factor contributing to the observed gender disparity may be a higher health awareness level among young women rather than men [43].

We are aware that our research may have some limitations. First, since our subjects were only urban residents in Tehran, our findings cannot be extrapolated to the entire country’s population. Second, in order to reach solid conclusions about the association of the “time period” with BMI and WC, more measurements and longer follow-up periods are needed. Third, the body composition of the participants was not recorded in our study; therefore, we could not speculate to what degree the reported weight fluctuations might correspond to body fat percentage changes. Moreover, we did not consider the role of the participants’ dietary intake and nutritional habits on their BMI and WC. However, the current study has several strengths worth mentioning. Anthropometric measurements were performed by trained staff instead of relying on self-reports, increasing the accuracy of measurements. Moreover, our research included participants from a wide adult age spectrum and birth cohorts, who were tracked for 15 years and underwent three measurement intervals.

In conclusion, this study demonstrated rising age-dependent trends in the BMI and WC of Tehranian adult men and women. However, there were substantial gender-specific associations with birth cohorts, indicating that the men born in recent birth cohorts and the women born in earlier birth cohorts had the most alarming trends in BMI and WC. Given Iran’s epidemiological transition state, it seems that the nation has a heterogeneous tendency for weight gain, and our findings suggest that effective population-based obesity preventive measures should be targeted towards gender, age, and birth cohort, as the most potent determinants of obesity.

## Supplementary Information

**Additional file 1.**

## Data Availability

The datasets used and analyzed during the current study are available from the corresponding author on reasonable request.
