# Supplementary material for: The association of the age, period, and birth cohort with 15-year changes in body mass index and waist circumference in adults: Tehran lipid and glucose study (TLGS)
Source: BMC Public Health. 2022 Mar 2;22:418. doi: 10.1186/s12889-022-12810-z (PMC8889713; doi:10.1186/s12889-022-12810-z)
Supplement: Supplementary file 1 — Additional file 1. [file 12889_2022_12810_MOESM1_ESM.docx]

| **Supplementary Table 1:** Baseline characteristics of respondents and non-respondents participants. | | | | |
| --- | --- | --- | --- | --- |
|  | | **Respondents (N=4895)** | **Non-respondents (N=5133)** | ***P*-value** |
| **Sex** (women) | | 2871 (58.7) | 2849 (55.5) | 0.001 |
| **Age** ( year) | | 41.4 ± 12.9 | 44.0 ± 16.6 | <0.001 |
| **Age groups** (year) n (%) | |  |  |  |
| **Men**  **N=4308** | 20-29 | 371 (18.3) | 484 (21.2) | <0.001 |
|  | 30-39 | 576 (28.5) | 525 (23.0) |  |
|  | 40-49 | 442 (21.8) | 378 (16.5) |  |
|  | 50-59 | 336 (16.6) | 300 (13.1) |  |
|  | 60-69 | 250 (12.4) | 389 (17.0) |  |
|  | ≥70 | 49 (2.4) | 208 (9.1) |  |
| **Women**  **N=5720** | 20-29 | 618 (21.5) | 745 (26.1) | <0.001 |
|  | 30-39 | 816 (28.4) | 647 (22.7) |  |
|  | 40-49 | 706 (24.6) | 439 (15.4) |  |
|  | 50-59 | 496 (17.3) | 433 (15.2) |  |
|  | 60-69 | 215 (7.5) | 424 (14.9) |  |
|  | ≥70 | 20 (0.7) | 161 (5.7) |  |
| **Weight** (kg) | | 70.7 ± 12.7 | 69.8±13.4 | <0.001 |
| **Waist circumference** (cm) | | 87.9 ± 11.9 | 88.0 ± 12.7 | 0.743 |
| **BMI** ( kg/m^2^) | | 26.9 ± 4.5 | 26.6 ± 4.9 | 0.002 |
| **Education** n (%) | |  |  | 0.066 |
|  | ≤12 years | 4518 (88.4) | 4255 (87.2) |  |
|  | >12years | 593 (11.6) | 625 (12.8) |  |
| **Physical activity** n (%) | |  |  | 0.886 |
|  | Active | 1197 (24.2) | 1168 (24.1) |  |
|  | Non active | 3752 (75.8) | 3686 (75.9) |  |
| **Marriage status** n (%) | |  |  | <0.001 |
|  | Single | 839 (16.3) | 600 (12.3) |  |
|  | Married | 4294 (83.7) | 4295 (87.7) |  |
| **Smoker** n (%) | | 564 (11.6) | 789 (15.9) | <0.001 |
| BMI, body mass index | | | | |
